# Spatially dynamic binaural cues have a larger influence on auditory stream segregation than static cues

**DOI:** 10.1038/s41598-026-52045-1

**Published:** 2026-05-11

**Authors:** Nathan C. Higgins, Carrie A. Secor, Erol J. Ozmeral

**Affiliations:** Department of Communication Sciences and Disorders, University of South Florida, Tampa, FL USA

**Keywords:** Auditory stream segregation, Bistable perception, Binaural cues, Perceptual hysteresis, Auditory scene analysis, Neuroscience, Psychology, Psychology

## Abstract

Auditory scene analysis is the process used to separate and integrate acoustic information into representations of auditory objects. Spatial cues play a critical role in this process, but questions remain regarding how these cues influence auditory stream segregation. The ABA stream segregation paradigm capitalizes on a bistable stimulus capable of eliciting perception of a single (integrated) auditory stream, or two separate (segregated) auditory streams with equal probability. Here, perceptual bistability was used to assess the effectiveness of interaural level (ILD), time (ITD), and correlation (IAC) values in promoting stream segregation when kept static versus dynamically modulated, across extended ABA sequences (100 + sec). Results showed that intermediately lateralized static binaural cues (7 dB, ILD; 225 µs, ITD) minimally affected stream segregation; in contrast, systematically modulated cues *did*, with perceptual hysteresis-like effects. These results show that perception of movement increases the influence of binaural cues as a stimulus feature for separating sound sources more than static cues. Comparison of binaural cue segregation boundaries and traditional discrimination thresholds showed that individuals with high sensitivity to a cue (i.e., low thresholds) were more likely to have narrower segregation boundaries, indicating common cognitive processes that support spatial stream segregation and sound localization.

## Introduction

To localize sounds in the horizontal plane, listeners rely on binaural cues, specifically small differences in time, level, and sound correlation between the two ears. As individuals navigate their surroundings, these cues provide a continuous flow of spatial information that the central auditory system uses, in combination with other sound features, to fuse acoustic features into integrated auditory objects, or to separate features into discrete, segregated auditory objects. This process, referred to as auditory stream segregation^1^, supports the multisensory spatial awareness that listeners rely on to monitor their surroundings across a full spectrum of environments, such as monitoring footsteps, vehicle-traffic, or listening to a specific talker in a loud restaurant.

*Primitive stream segregation*, as termed by Bregman^1^, happens automatically in a bottom-up process based on differences in stimulus properties such as frequency, repetition rate, and spatial cues that allow the auditory system to group or separate features to form auditory objects. The ABA stream segregation paradigm provides a well-established basis for studying primitive streaming mechanisms. In the traditional experimental setup, tones are presented in a low-high-low-blank (ABA_) sequence referred to as triplets, where the A and B represent tones of different frequency, and the underscore a missing B-component. The degree of frequency separation and the presentation rate of the tones determines the proportion of time that a listener perceives (1) a single-integrated auditory stream described as galloping, or (2) two segregated auditory objects described as two metronomes^1–3^. Larger frequency separations, such as ten semitones, are disproportionately perceived as segregated, with smaller frequency separations, such as 3 semitones, as an integrated percept^4^. An intermediate separation of six semitones, with an inter-triplet interval around 500 ms, is considered bistable, meaning that upon repeated presentation (20 + sec), listeners will typically switch back and forth spontaneously between the two percepts, with equal probability of reporting an integrated or segregated percept at any given time, following an initial period of integration^5–8^. While this approach relies on a subjective measure of participants’ willingness and ability to consciously assign a perceptual state to their moment-to-moment experience, the benefit is that the switch from segregated to integrated or integrated to segregated represents a cognitive process primed for exploring the stimulus parameters that drive perception^1,3^. Illustrating this phenomenon, Byrne et al.^9^ dynamically manipulated the frequency separation of the A- and B-components of ABA triplets (centered at 5 semitones) with modulation times of 5, 10, or 20 s per cycle. Despite using a range of frequency separation that typically results in bistable or biased towards integration (floor to ceiling: 3.5 to 6.5 semitones), they observed clear patterns of perceptual switching that matched the rate of changing frequency separation, indicating that even small modulations of a stimulus parameter influence perception differently compared to static cues^1,3,10^.

While the role of binaural spatial cues in auditory stream segregation is still being explored, psychoacousticians have thoroughly characterized binaural cues with respect to discrimination thresholds^11,12^, their role in sound localization^13–16^, and their capacity to provide spatial release from masking to improve speech intelligibility^17–21^. Studies have also used binaural cues to investigate how the auditory system separates sound sources^22–24^. Hartmann and Johnson^22^ for example, asked listeners to identify two melodies interleaved on A and B streams, respectively, while testing conditions with different peripheral channels, i.e., A and B streams separated by frequency or binaural cue. Results showed that separation of the A and B by an octave, or by ear of presentation (i.e., dichotic listening) was sufficient for the auditory system to separate and identify both melodies. When the A and B were separated by interaural time (ITD, 500 µs) and interaural level differences (ILD, 8 dB), listeners also showed better performance than the control diotic-condition, though with a weaker effect than the octave and dichotic conditions^22^. In a similar experiment in the free field, Middlebrooks and Onsan^23^ presented two non-temporally overlapping streams (1-target, 1-masker) and measured the spatial separation necessary to identify which target (of two possible) was present^23^. Rhythmic masking release thresholds were then compared to minimum audible angle measures in the same participants. Surprisingly, no clear relationship was observed between these two thresholds, interpreted as evidence that spatial stream segregation and sound localization may rely on different processing mechanisms^23^. These experiments represent examples of *schema-based* stream segregation, as they require familiarity with a sound pattern or melody to do the task^22,23^, rely on top-down volitional control of perception^1^, and are used to quantify the empirical limitations of listeners’ sensitivity to small stimulus differences.

Additional studies quantifying the effect of binaural cues on stream segregation using subjective self-reports have shown mixed results. Anstis and Saida^5^, showed that segregated percepts tended to be “reset” to integrated when ABABs were initially presented monaurally but then suddenly switched to the other ear^5^. Bregman and Rogers in a series of experiments, showed similar effects when ABA triplets were abruptly shifted in lateralized space via ILD or ITD cues, consistent with a free field version of the experiment^25,26^, though this effect was somewhat lessened when the spatial cue slowly adapted to the final “test” location. Results from Boehnke and Phillips^27^ showed strong segregation when the A and B were presented to opposite ears (i.e., monaural) and for the ILD condition, but relatively poor segregation for the ITD and the varying loudness conditions^27^.

In contrast Haywood et al.^28^ showed a significant effect of ITD on segregation by manipulation of interaural phase delays and Füllgrabe and Moore^29^ showed a significant increase in segregation due to ITD on the A and B components compared to a diotic control-condition. Taken together, these studies show that sudden shifts in lateralization disrupt segregation and stable cues can promote segregation, but none of them address how modulated dynamic cues affect stream segregation (e.g., Byrne et al.^9^).

To further investigate these questions, Experiment 1 tested the hypothesis that static binaural cues (ILD and ITD) enhance auditory stream segregation compared to a bistable diotic condition with the expectation of observing a baseline increase of segregation to contrast with the diotic control-condition and dynamically modulated cues. Experiment 2 tested the hypothesis that dynamically modulated ILD, ITD, and IAC (interaural correlation) cues, by providing greater saliency, effectively promote and inhibit stream segregation as a function of the modulation. Finally, participant response functions from the dynamic cue modulation were used to estimate a binaural cue segregation boundary, which was then compared to traditional measures of binaural cue discrimination to test the hypothesis that the same cognitive processes underly spatial stream segregation and sound lateralization.

## Experiment 1: static ILD and ITD in a bistable ABA paradigm

### Methods

#### Participants

Fifteen human participants (12 female) in the age range of 19 to 28 (mean: 21.1 *±* 2.5 years) were recruited for this study. All participants had normal audiometric evaluations with pure tone thresholds ≤ 25 dB hearing level at octave frequencies between 250 Hz and 8 kHz and ≤ 10 dB asymmetry between 1 and 6 kHz. All procedures were approved by the University of South Florida Institutional Review Board and all procedures were performed in accordance with the approved guidelines and regulations. Participants provided written informed consent and were paid for their participation.

#### Equipment and environment

During testing, participants were seated in a double-walled sound attenuation booth. Acoustic stimuli were delivered over ER-2 Etymotic insert earphones. Participant responses were collected with a 4-key USB response pad (XK-4 USB Stick, P.I. Engineering, MI) for the ABA stream segregation task. All participant responses, and all stimuli were made and delivered via Matlab (The MathWorks, Inc., Natick, MA) software.

#### Procedure and stimuli: ABA stream segregation

**Fig. 1:**
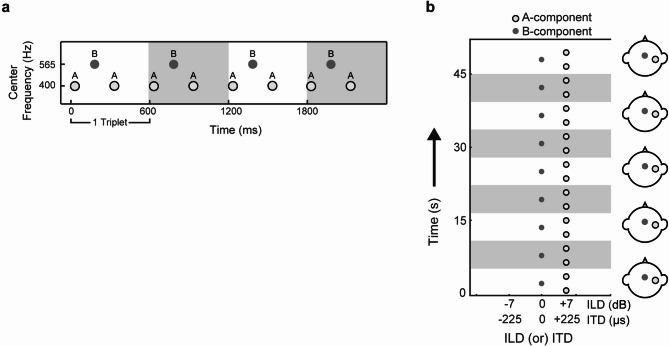
(**a**) Schematic of diotic bistable stimulus used in both Experiments 1 and 2. Gray circles (narrowband noisebursts) correspond to A (light gray) and B (dark gray) components of the ABA triplets. Horizontal background shading illustrates separation of each triplet. (**b**) Schematic of static binaural cue ABA paradigm used in Experiment 1, where the B component was diotic (ILD = 0 dB, ITD = 0 µs) while the A-components were presented at + 7 dB ILD or + 225 µs ITD, respectively. A (light gray) and B (dark gray) components represented by circles, vertical background shading illustrates separation of ABA triplets. Head schematic represents the evoked lateralized percept over time.

All ABA triplets presented in this study had similar temporal components, as illustrated in Fig. 1a. The full triplet can be presented with the notation: ABA_ where the “_” corresponds to a missing B-component. Each of the A (Fig. 1a, light grey circles) and B (Fig. 1a, dark grey circles) components were 50 ms duration with a 10 ms onset and offset cosign-shaped ramp, and a 150 ms interstimulus interval between the onset of each A- and B-component, for a total duration of 600 ms for each triplet. In Experiment 1, all triplets were composed of narrow band noise bursts with a center frequency of 400 Hz for the A- and 565 Hz for B-components each with a 50 Hz bandwidth. Prior to the onset of each trial, A and B noise tokens were generated and used for all triplets within that trial (i.e., frozen noise), where all A-components were identical noise tokens, the B-components were identical noise tokens, but they were different from one another. Note that the frozen noise tokens were different for each trial. The choice of frozen noise tokens was made to minimize perceptual segregation based on changing spectral-temporal dynamics from triplet-to-triplet. All acoustic stimuli were generated at a sampling rate of 96 kHz.

The stimulus parameters used in this experiment were selected with the goal of providing a basis for carrying binaural cues that will be perceptually lateralized, i.e., narrowband noise bursts rather than pure tones^30^, while also avoiding disruption of the bistable perceptual dynamics typically observed with a 6-semitone frequency separation.

#### Bistable diotic condition

In Experiment 1, each trial of the diotic bistable condition consisted of 174 triplets presented in sequence for a total duration of 104.4 s (174 triplets *×* 0.6 s/triplet = 104.4 s). All participants went through a familiarization process with bistable stimuli to ensure confidence interpreting their perception as one or two streams and reporting it via the two-button continuous response paradigm, referred to by Bregman (1990) as a measurement of the “proportion of time integrated and segregated”. Participants were instructed that, “There are no right or wrong answers; we just want to know which percept you are experiencing”. During familiarization, examples of ABA triplets with large (9 semitones) and small frequency separation (3 semitones) were presented in addition to multiple practice trials (2 to 3) of the experimental bistable paradigm (6 semitones). A diotic condition trial was always presented first to ensure familiarity with the bistable percept. After that, all trials (including additional diotic conditions) were randomly presented. Participants completed at least 3 trials of all conditions.

#### Static ILD and ITD conditions

In Experiment 1, each ILD and ITD trial consisted of 174 triplets presented in sequence (104.4 s total duration). In the static ILD trials, the B (Fig. 1b, dark grey circles) component was diotic while the A (Fig. 1b, light grey circles) components were at + 7 dB ILD, lateralized to the right of the midline. During the static ITD trials, the B-component was diotic while the A-component was set at + 225 µs ITD, also lateralized to the right of the midline. These values of ILD and ITD were chosen because they represent an intermediate but easily lateralized cue^12,31^ for narrowband noise bursts in this frequency range (400 Hz center-frequency). Participants were instructed to continuously report their perception on the response pad; three trials of each static ILD and static ITD condition were collected.

#### Data analysis

Response time-courses were recreated based on button press time stamps and provided the basis for all subsequent stream segregation analysis. Using a sample-size of 100 ms from the onset of the first ABA triplet through the end of the trial, every sample was designated as an integrated or segregated percept as a function of the prior button press. Initial samples in the trial prior to the first button press were designated by the first button press. Trials with two or less switches were excluded from further analysis. To test for an effect of binaural cue, the perceptual probability averaged over time for each participant, for each condition, was input to a repeated measures ANOVA with an alpha threshold of 0.05. A Bayesian paired samples t-test using Markov Chain Monte Carlo (MCMC) sampling (5000 iterations, burn-in period of 1000) was used as a follow-up evaluation comparing each binaural cue and the diotic condition.

### Results

**Fig. 2:**
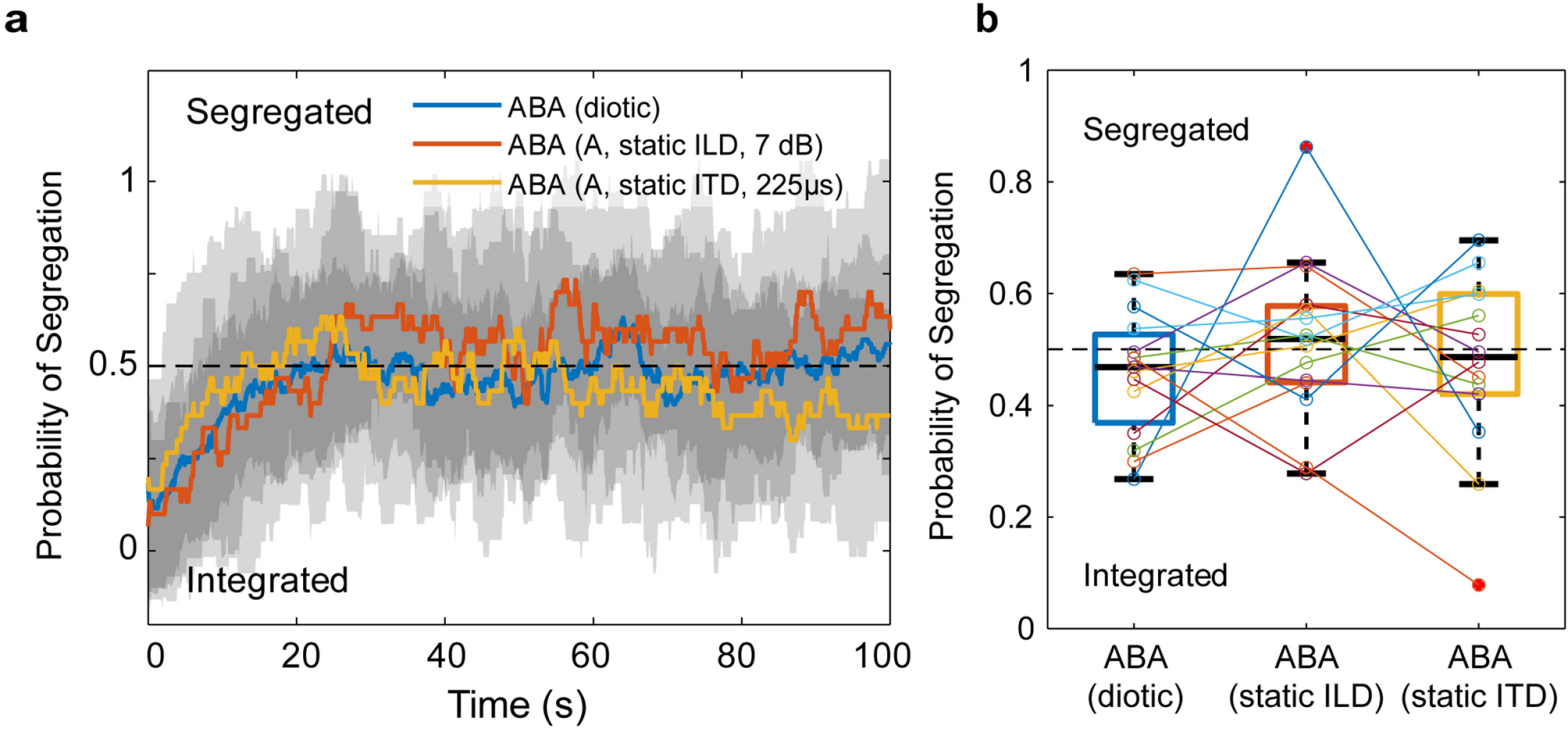
Experiment 1, static binaural cue responses. (**a**) Response patterns for the static ILD (orange line) and ITD (yellow line) conditions showed approximately equal proportion of integrated and segregated perception, the same as the diotic conditions (blue line). Shading corresponds to standard deviation. (**b**) Box plots for each condition illustrate the relative variability of the subject responses across condition.

The response patterns representing the influence of static binaural cues are shown in Fig. 2. During the bistable diotic condition (Fig. 2a, blue line), participants on average demonstrated clear features of bistable perception. Following an initial integrated percept, beyond 10 s the probability of a segregated percept rose to 0.5 and the group average remained there for the rest of the trial. The static ILD condition (Fig. 2a, orange line) elicited similar average perception over time despite a lateralized separation of the A- and B-components of the ABA (Fig. 1b); initially integrated followed by approximately equal proportion of the integrated and segregated percepts with no effect of ILD. During the ITD condition (Fig. 2a, yellow line), participant response patterns similarly demonstrate bistable perception, though the group average toward the later parts of the trial indicate slightly more integrated than segregated perception. The overall average probability (Fig. 2b) of the diotic, static ILD and ITD cues to influence stream segregation was tested using a repeated measures ANOVA. No significant main effect was observed between the conditions (F_2,26_ = 0.653, *p* = 0.53, η^2^*p* = 0.048). Follow-up testing using Bayesian paired samples analysis, while indicating the potential of a weak relationship, also supported the null hypothesis of no effect (mean difference of 0.059 (95% CI [−0.04, 0.19]), with a posterior probability that static ILD condition was greater than diotic of P(µ_diff_ > 0) = 0.89. Similarly, no effect in mean probabilities was observed between the diotic and static ITD conditions (mean difference = −0.018, 95% CI [−0.11, 0.081], P(µ_diff_ > 0) = 0.34).

### Discussion

The results presented here demonstrate the effectiveness of the ABA paradigm with a 6-semitone center-frequency separation using narrowband noise bursts to elicit equal proportions of an integrated and segregated percept over time (Fig. 2, blue line). Despite the clear consistency observed in the group average, high variability was observed over time, leading to the hypothesis that shifting the A-component of the ABA triplets laterally, via ILD (+ 7 dB) or ITD (+ 225 µs) manipulation, would shift perception by a measurable amount towards more segregated than integrated^29^. Results of the static ILD (Fig. 2, orange line) and ITD (Fig. 2, yellow line) conditions counter that expectation, instead showing little to no influence on perception compared to the diotic, control condition.

**Fig. 3:**
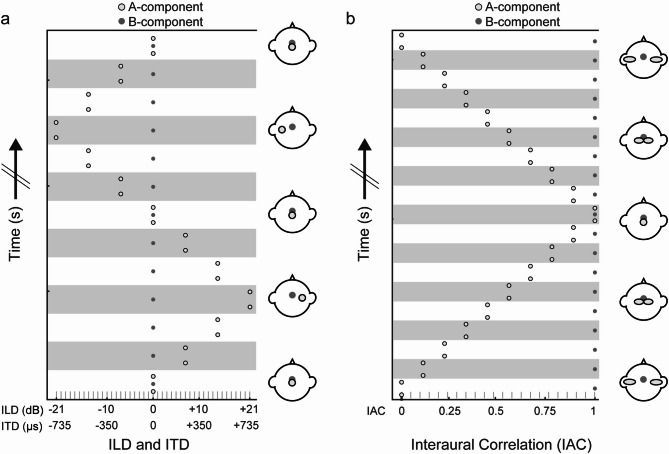
Experiment 2, schematics of the modulated binaural cue ABA paradigm for ILD and ITD (**a**) and IAC (**b**) conditions. Gray circles correspond to A (light gray) and B (dark gray) components of the ABA triplets. Vertical background shading illustrates separation of each triplet. Note that the y-axis (time) is not continuous as there were multiple binaural cue steps between displayed triplet; notches on the x-axis represent each binaural cue step. Diagrams on the right side of A and B represent the lateralized percept over time.

Previous studies that tested the effects of dichotic ABAB-type stimuli on stream segregation show mixed results depending on the paradigm and stimulus parameters^3,5,25,26^. Boehnke and Phillips^27^, for example, individually manipulated A and B noise bursts as a function of ILD (± 12 dB), ITD (± 600 µs), overall level, and ear of presentation to test the effect on stream segregation. Results showed a strong influence towards a segregated percept for the monaural ear of presentation condition (same as Van Noorden^3^ and ILD condition, and a weak influence of ITD and overall level (i.e., both led to a greater proportion of integrated percept). Methodological factors that are the likely reason for the difference in results: the present study (1) had a 6-semitone center-frequency separation between the narrowband noise bursts, (2) used triplet sequences presented for over 100 s duration, and (3) applied ILD and ITD cues to only the A-components of each triplet while the B-component was diotic. In contrast, Boehnke and Phillips used broadband noise bursts with no frequency separation between the A- and B-components, triplets were presented for less than 15 s (similar to the first 15 s, Fig. 3, orange and yellow lines), and ILD and ITD cues were applied to both A and B-components of each triplet.

Additional evidence that static binaural cues promote segregation comes from studies using an objective streaming paradigm where detection of anisochronous components of ABAB sequences is easier when the listener experiences an integrated percept^24,29,32,33^. In this objective streaming task^34^, increasingly larger ITDs “obligate” the listener to perceive segregation, making the detection of the anisochronous target sequence more difficult and increasing reaction time threshold, establishing evidence that static ITDs and ILDs^33^ promote segregation. In addition to paradigm differences (obligatory versus subjective segregation), stimulus differences provide another explanation for differing results. David et al.^33^ used speech shaped noise-bursts (approximately flat spectrum from 0 to 1000 Hz), stimuli that are more likely to convey ITDs and ILDs as a lateralized percept leading to more segregation compared to the narrowband noise bursts used in the current study.

Füllgrabe and Moore^29^, in an experiment very similar to the one presented here, measured the proportion of segregation over 30 s ABA sequences and observed a significant effect of static ITDs at physiologically relevant levels of 250, 300, 400, and 500 µs. With respect to those results^29^, the lack of segregation observed for comparable ITDs in the current study using narrowband noise bursts rather than pure tones, is surprising. One likely explanation is in the composition of the ABA triplets. Same as in Boehnke and Phillips^27^, ITDs were applied to both the A and B-components of each triplet^29^, likely to evoke the perception of a sound jumping from left to right. In this manner, a 250 µs ITD would have the A at −250 µs ITD and the B at + 250 µs ITD, creating a cross-hemifield jump in lateralization. In our study (using 250 µs ITD as an example), that would manifest with the A at + 250 µs ITD and the B at 0 µs ITD, which is a midline to lateral jump, and half the difference between the two components. Psychophysically, listeners consistently demonstrate the best spatial resolution around the midline^31,35,36^, and these differing binaural cue triplet-configurations are likely to trigger different spatial channels^37,38^. Overall, the evidence from this study and previous studies indicates that static binaural cues provide a usable cue for objective, volitional stream segregation, but are a relatively weak cue when active spatial listening is not the primary objective.

## Experiment 2: modulated ILD, ITD, and IAC in a bistable ABA paradigm

### Methods

#### Participants

Thirty-one (18 female) human participants in the age range of 18 to 35 (mean: 23.58, *±* 4.48) years were recruited for this study. One individual overlapped with Experiment 1. All participants had normal audiometric evaluations with pure tone thresholds ≤ 25 dB hearing level at octave frequencies between 250 Hz and 8 kHz and ≤ 10 dB asymmetry between 1 and 6 kHz. All procedures were approved by the University of South Florida Institutional Review Board and performed in accordance with the approved guidelines and regulations. Participants provided written informed consent and were paid for their participation.

#### Equipment and environment

During testing, participants were seated in a double-walled sound attenuation booth. Acoustic stimuli were delivered over ER-2 Etymotic insert earphones. Participant responses were collected with a touchscreen monitor for the binaural cue discrimination threshold task and with a 4-key USB response pad (XK-4 USB Stick, P.I. Engineering, MI) for the ABA stream segregation task. During the ABA stream segregation conditions, electroencephalography data (not reported here) was collected, participants were instructed to remain still and attempt to limit eye blinks. All participant responses, and all stimuli were made and delivered via Matlab (The MathWorks, Inc., Natick, MA) software.

#### Procedure and stimuli: binaural cue discrimination thresholds

Binaural cue discrimination thresholds for ILD, ITD, and IAC were measured with a 4 interval, 2-alternative forced choice paradigm^12^ using a 2-down, 1-up, adaptive procedure that estimated a 70.7% accuracy^39^. Stimuli were generated in part using the binaural toolbox (Akeroyd, 2017). Intervals 1 and 4 were the diotic reference, while interval-2 or interval-3 represented the target with the tested binaural cue. Participants were instructed to select the interval (2 or 3) that was different from the other three and indicate their answer on a touchscreen monitor. For ILD and ITD tracks, that translates to a percept that the 2nd or 3rd interval is lateralized relative to the other three. For IAC the perception is that the 2nd or 3rd interval is wider or more diffuse than the other three.

Stimuli consisted of a 300 ms narrowband noise burst and a 15 ms rise/fall time generated with a sampling frequency of 96 kHz. Noise burst intervals had a center frequency of 500 Hz (1/3rd octave bandwidth), with a 500 ms silent interstimulus interval. An additional condition also tested ILD discrimination thresholds using narrowband noise burst intervals with a 4000 Hz center frequency (1/3rd octave bandwidth). All sounds were presented at 70 dB SPL for ITD and IAC conditions. ILD conditions were presented with an average binaural level of 70 dB SPL, however, to avoid the potential confound that a participant may use monaural sound level cues to detect the target interval, a random value selected from the range of ± 5 dB was applied to each of the four intervals^12^.

IAC targets were imposed on test intervals by mixing two independently generated bandpass filtered noise bursts:

$$\:LeftEar=\:\sqrt{1+IAC}\:\times\:Noise1+\:\sqrt{1-IAC}\:\times\:Noise2$$

$$\:RightEar=\:\sqrt{1+IAC}\:\times\:Noise1-\:\sqrt{1-IAC}\:\times\:Noise2$$

The IAC term (alternately termed ρ) represents the interaural correlation (see:^40^). To ensure the final IAC value matched the desired value, a library of 100 noise-token pairs with verified IAC levels were pre-generated and stored. During IAC experimental trials, the appropriate noise tokens were randomly selected from the library and used as the IAC target.

Each trial ended once 12 reversals on the adaptive track were achieved. The average binaural cue levels for the last six reversals were taken as the threshold for that trial; each adaptive track was equivalent to a trial. Adaptive tracks had an initial starting value of 8 dB ILD, 1400 µs ITD, or 0.85 for IAC. For ILDs and ITDs the adaptive track progressed by a factor of two over the first 4 reversals, and then a factor of $\:\sqrt{2}$ for reversals 5 to 12. For IAC conditions the steps changed by 0.0486 over the first 4 reversals and 0.0282 for reversals 5 to 12. Three thresholds were collected for each condition, the average of the three represented the discrimination threshold for that participant for that binaural cue. In total there were 4 conditions, ILD-low frequency, ILD-high frequency, ITD, and IAC.

#### Procedure and stimuli: ABA stream segregation

All ABA triplets had similar temporal components to Experiment 1, illustrated in the Fig. 1a schematic (see additional detail in Experiment 1 above). In Experiment 2, except for the high frequency ILD condition, all triplets with a frequency separation were composed of narrow band noise bursts with a center frequency of 400 Hz for the A- and 565 Hz for the B-component with a 1/3rd octave bandwidth. This frequency separation of A- and B-components (400 and 565 Hz) approximates a pure-tone 6-semitone separation (e.g.^6^) and will be referred to as *low-frequency*,* with frequency separation* conditions. There was an additional ILD condition where the A-component was 4000 Hz and the B was 5650 Hz, also a 6-semitone frequency separation; termed *high-frequency*,* with frequency separation*. In conditions without a frequency separation, the B-component had a center frequency that matched the A-component (400 Hz for low-frequency, 4000 Hz for high-frequency). These are termed *low-* (or) *high-frequency*,* no frequency separation* conditions. Participants were presented at least three full trials of every condition. As in Experiment 1, triplets were composed of separate frozen noise tokens for the A- and B-components in each trial.

##### Modulated ILD and ITD conditions

In addition to the diotic control condition (identical to Experiment 1), Experiment 2 tested dichotic conditions in which ILD or ITD cues were systematically modulated on the A-components (Fig. 3a, dark grey circles) of each triplet while the B-component (Fig. 3a, light grey circles) was diotic. The A-component ILD cues were modulated at a rate of 1 dB per triplet over the range of −21 dB ILD to + 21 dB ILD, generating a dynamically moving spatial trajectory. Each trial began with the A-components at the midline (0 dB, ILD), progressing laterally to the right (+ 21 dB, ILD), proceeding in the opposite direction laterally to the left (−21 dB, ILD), then returning to the midline (0 dB ILD) to complete one full cycle, representing 87 triplets. Two full cycles were presented per modulated-ILD trial for a total of 174 triplets, and 104.4 s total duration for each trial.

Similarly, in separate trials, ITD cues were systematically modulated on the A-components (Fig. 3a, dark grey circles) of each triplet while the B-component (Fig. 3a, light grey circles) was diotic. The A-component ITD cues were modulated at a rate of 35 µs per triplet over the range of −700 µs ITD to + 700 µs ITD, generating a dynamically moving spatial trajectory. Each trial began with the A-components at the midline (0 µs, ITD), progressing laterally to the right (+ 700 µs, ITD), proceeding in the opposite direction laterally to the left (−700 µs, ITD), then returning to the midline (0 µs, ITD) to complete one full cycle, representing 87 triplets. Two full cycles were presented per modulated-ILD trial for a total of 174 triplets, and 104.4 s total duration for each trial.

##### Modulated IAC condition

In the modulated IAC condition, IAC cues were systematically modulated on the A-components (Fig. 3b, light grey circles) of each triplet while the B-component (Fig. 3b, dark grey circles) was diotic. IAC cues on the A-components were modulated at a rate of 0.0486 over the range of 0 to 1 IAC, generating a dynamically changing trajectory. Each trial began with the A-components at 0 IAC, proceeded to converge towards an IAC of 1, and then cycled back to an IAC of 0. The step size bordering 1 had an IAC of 0.028^41^. Each cycle consisted of 43 triplets. Four cycles were presented per trial, every other cycle included an additional 10 triplets of IAC = 1 to provide additional samples of the correlated triplets. In total, each IAC trial had 190 triplets for 114 s total duration.

#### Data analysis

##### Stream segregation time-course

Response time-courses were recreated at 100 ms resolution based on button press time stamps, same as in Experiment 1 (see above for details). Trials with two or less switches were excluded from further analysis. Participant response functions were modeled using the fit.m function in Matlab with the ‘smoothingspline’ option and the formulas detailed below.

##### ILD and ITD binaural cue segregation boundary

To define the binaural cue segregation boundaries for each participant, ILD and ITD functions were calculated as the probability that a segregated percept was reported for each binaural cue level derived from the recreated button-response time course. The first 60 samples (6 s) were excluded to remove variability in how long it took for the initial button-press. The outcome of this analysis was a U-shape representing the probability of segregation as a function of binaural cue, this shape was then fit with an inverse Gaussian function:

$$\:{P\left(segregated\right)=h*{e}^{-\left(\frac{x-c}{w}\right)}}^{2}+s$$

where *h* represents the height of the peak, *c* the center, *w* the width, and *s* the vertical shift of the curve. For each participant the two points where the fit function reached the full width at half minimum were averaged together and used to define the binaural cue segregation boundary.

##### IAC binaural cue segregation boundary

For each participant, IAC functions were calculated as the probability that a segregated percept was reported as a function of each presented IAC cue. Response functions were then fit with an inverse logarithmic function:

$$\:P\left(segregated\right)=\:\frac{r}{\mathrm{log}\left(b*x\right)}+v$$

Where *r* represents the vertical range, *b* the base of the logarithm, and *v* the vertical offset of the curve. For IAC, the binaural cue segregation boundary was defined as the point 1/3rd of the range of the function, effectively describing the knee or transition point of the curve.

### Results

#### Response patterns

**Fig. 4:**
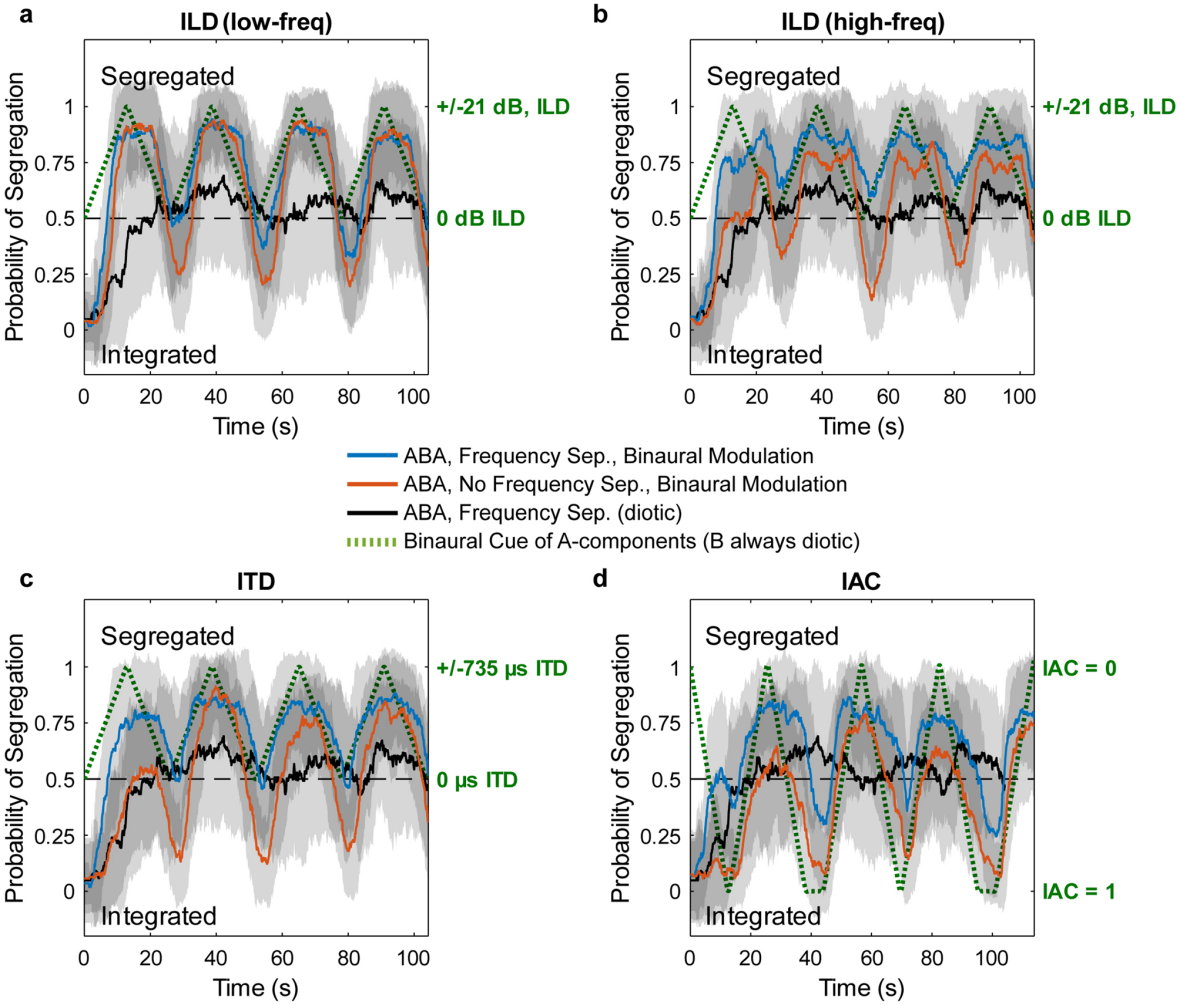
Experiment 2, response patterns for the modulated (**a**) ILD low-frequency, (**b**) ILD high-frequency, (**c**) ITD, and (**d**) IAC conditions. In each panel the dashed black line represents the 0.5 probability level, the solid black trace the diotic ABA control condition, the blue trace corresponds to the conditions *with* frequency separation, and the orange trace to the conditions *without* frequency separation. The dashed green lines represent the binaural modulation of the A-component over time for each respective cue over the range indicated by the right-side y-axis. Shaded error bars correspond to standard deviation.

**Fig. 5:**
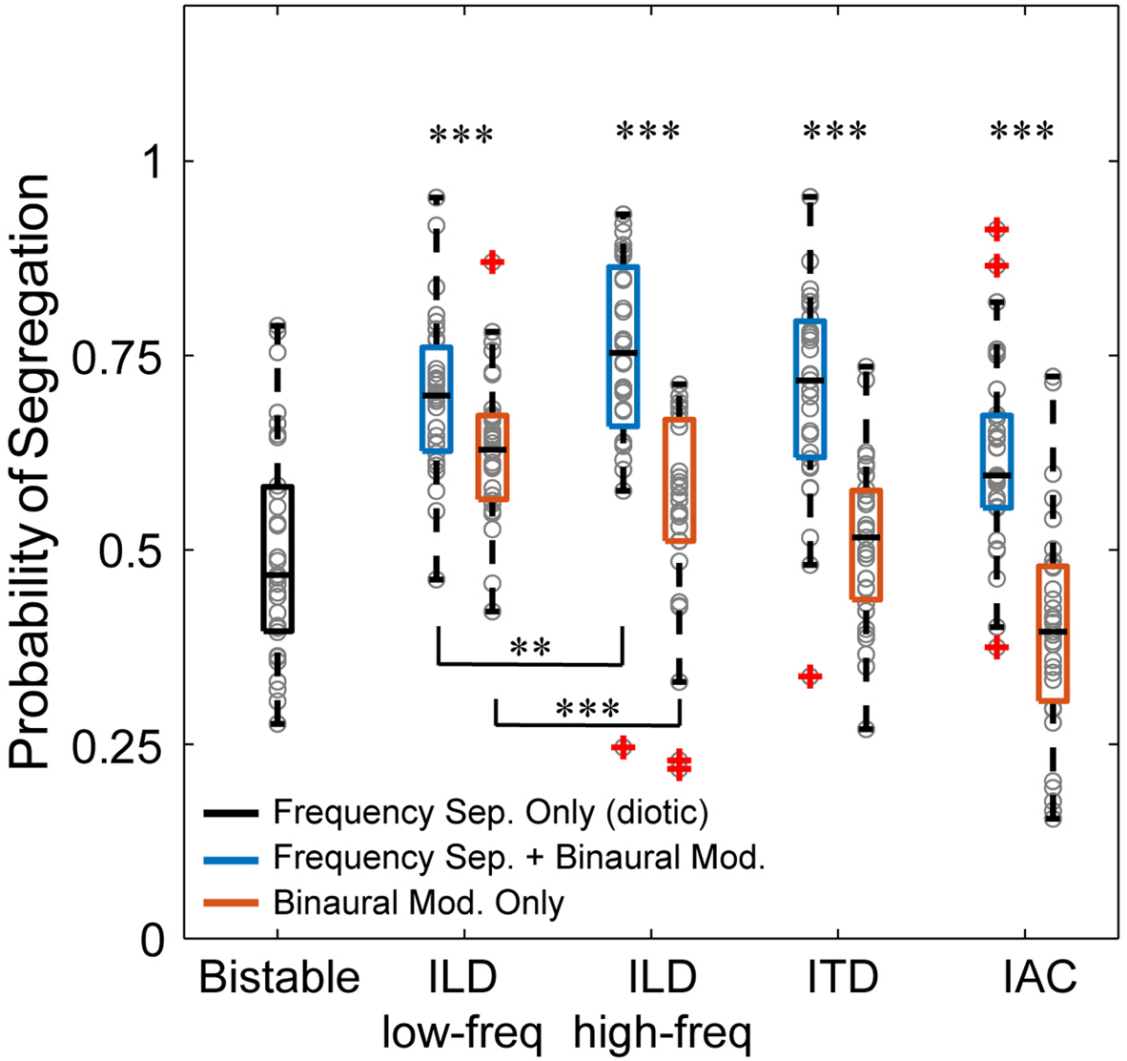
Box plots representing average proportion of the reported segregated percept for all conditions in Experiment 2. Open gray circles represent the data points for each participant. Outliers represented by red cross. Post hoc significance: ***p < 0.001, **p < 0.01.

Figure 4 shows the grand average response patterns for all eight conditions with the modulated binaural cues (Experiment 2). The baseline diotic condition exhibited the bistable nature of the stimuli without the influence of binaural cue modulation illustrated by the group average overlaid on each plot for comparison (Fig. 4; black line). Participant response patterns for all conditions with binaural cue modulation exhibited a strong influence of the modulation, demonstrated by an increase in segregation as perception followed the track of modulation on the A-component (Fig. 4, green lines, right y-axis) for all conditions with frequency separation (Fig. 4, blue lines) and without frequency separation (Fig. 4, orange lines). The main effects of perception with respect to frequency separation (two levels: with and without) and binaural cue condition (four levels: high and low frequency ILD, ITD, and IAC), were tested using a repeated measures ANOVA based on the average probability of segregation from the eight modulated binaural cue conditions (Fig. 5). Significant main effects for frequency separation (F_1,27_ = 137.97, *p* < 0.001, η^2^*p* = 0.84) and condition (F_3,81_ = 36.29, *p* < 0.001, η^2^*p* = 0.57) were observed, as well as an interaction of frequency by condition (F3,81 = 20.81, *p* < 0.001, η2p = 0.44). Rank-order post-hoc comparisons testing the effect of frequency separation showed the greatest effect was observed for IAC (t_30_ = 10.45, *p* < 0.001, d = 0.22) while the low-frequency ILD condition had the smallest (t_30_ = 3.88, *p* < 0.001, d = 0.067) effect, with the high frequency ILD and ITD conditions intermediate (*p* < 0.001 for both; Fig. 5). Comparison of the low-frequency ILD versus high-frequency ILD conditions showed an interaction for frequency separation versus without frequency separation. With separation, the high frequency condition elicited significantly more segregation (t_27_ = −2.83, *p* < 0.01, d = −0.057) than without (t_30_ = 3.71, *p* < 0.001, d = 0.075). This interaction can be seen in the Fig. 5 boxplots, where segregation increased for the high frequency ILD *with* separation, compared to the low frequency ILD *with* separation (blue box versus blue box), but segregation decreased for that contrast *without* frequency separation (orange box versus orange box).

#### Binaural cue segregation boundaries

**Fig. 6:**
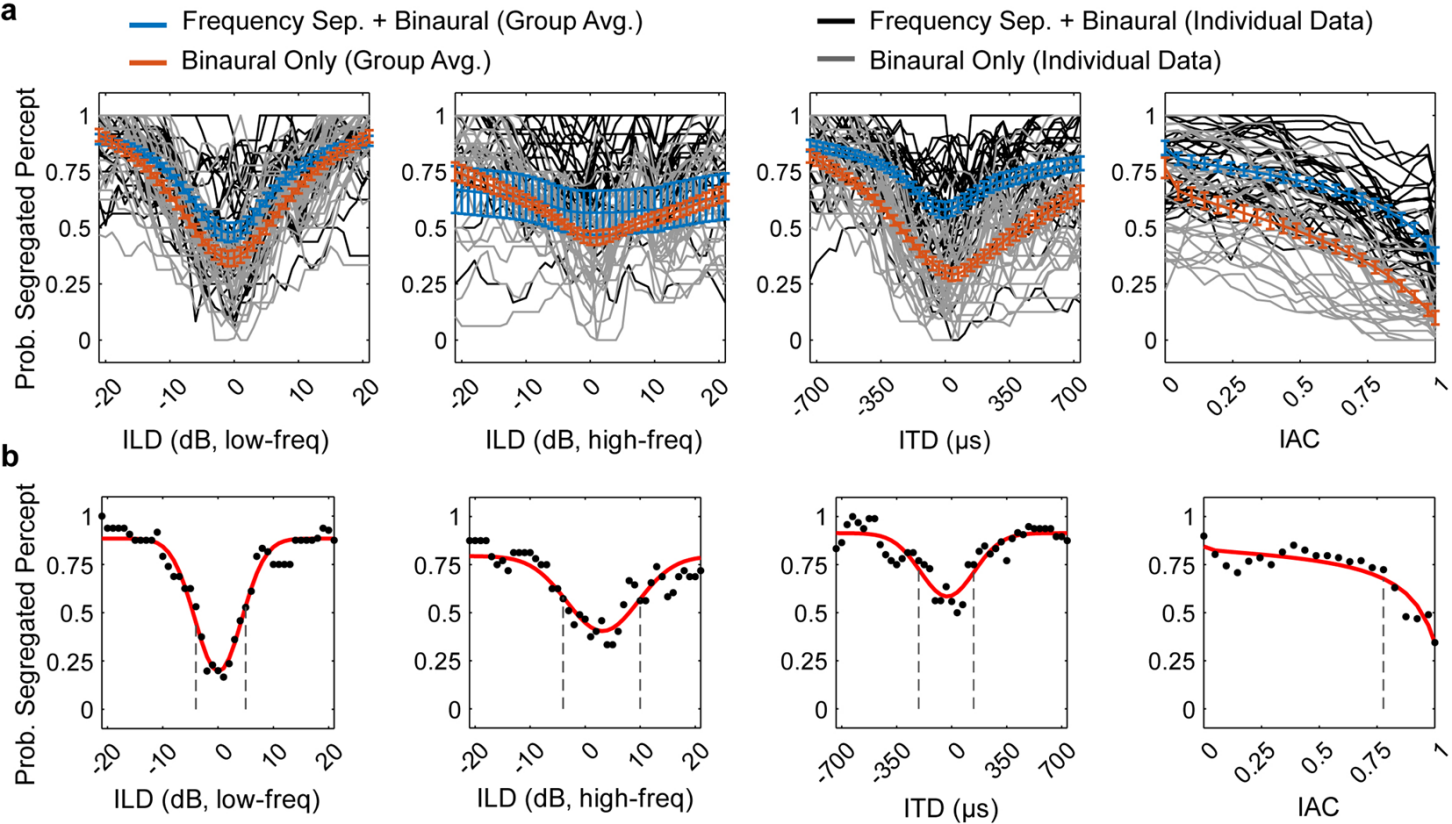
(**a**) Binaural cue response functions show the probability of a segregated percept for the low frequency ILD (panel 1), high-frequency ILD (panel 2), ITD (panel 3), and IAC (panel 4) conditions. In all panels the blue (group avg.) and black (individual participants) lines correspond to the with frequency separation condition, and the orange (group avg.) and gray (individual participants) lines without frequency separation. Error bars correspond to standard error of the mean. (**b**) Individual examples from four participants for each condition show the average probability at each binaural cue (black circles), respective fit function (red lines), and the defined segregation boundaries (vertical dashed black lines).

To calculate the binaural cue segregation boundary, a response function was generated for each participant representing the probability of perceiving a segregated percept with respect to each binaural cue in the modulation. Figure 6a presents the full modulated binaural cue dataset for individual participants plus group averages. Individual traces underline the high variability observed between individuals, with some exhibiting relatively flat modulation as a function of the binaural cue and others a strong U-shaped function. Also apparent are the effects of the binaural cue modulation with frequency separation (Fig. 6a, blue and black lines) compared to the binaural cue modulation without frequency separation (Fig. 6a, orange and grey lines). Figure 6b presents four examples of binaural cue response functions, each from a different participant and different condition. As illustrated in panels 1 to 3, for all ILD and ITD datasets an inverted gaussian function (Fig. 6b, red line) was fit to the data points (Fig. 6b, black dots) representing the response function for each respective participant. From the modeled response (Fig. 6b, red lines), the two points in binaural space corresponding to the full- width, half-min (Fig. 6b, black dashed lines, panels 1 to 3), were averaged to define the binaural cue segregation boundary. The datapoints for each participants’ IAC binaural cue functions were fit with an inverse log function, and the binaural cue segregation boundaries were defined by the elbow-point of the function (Fig. 6b, panel 4, black dashed line).

**Fig. 7:**
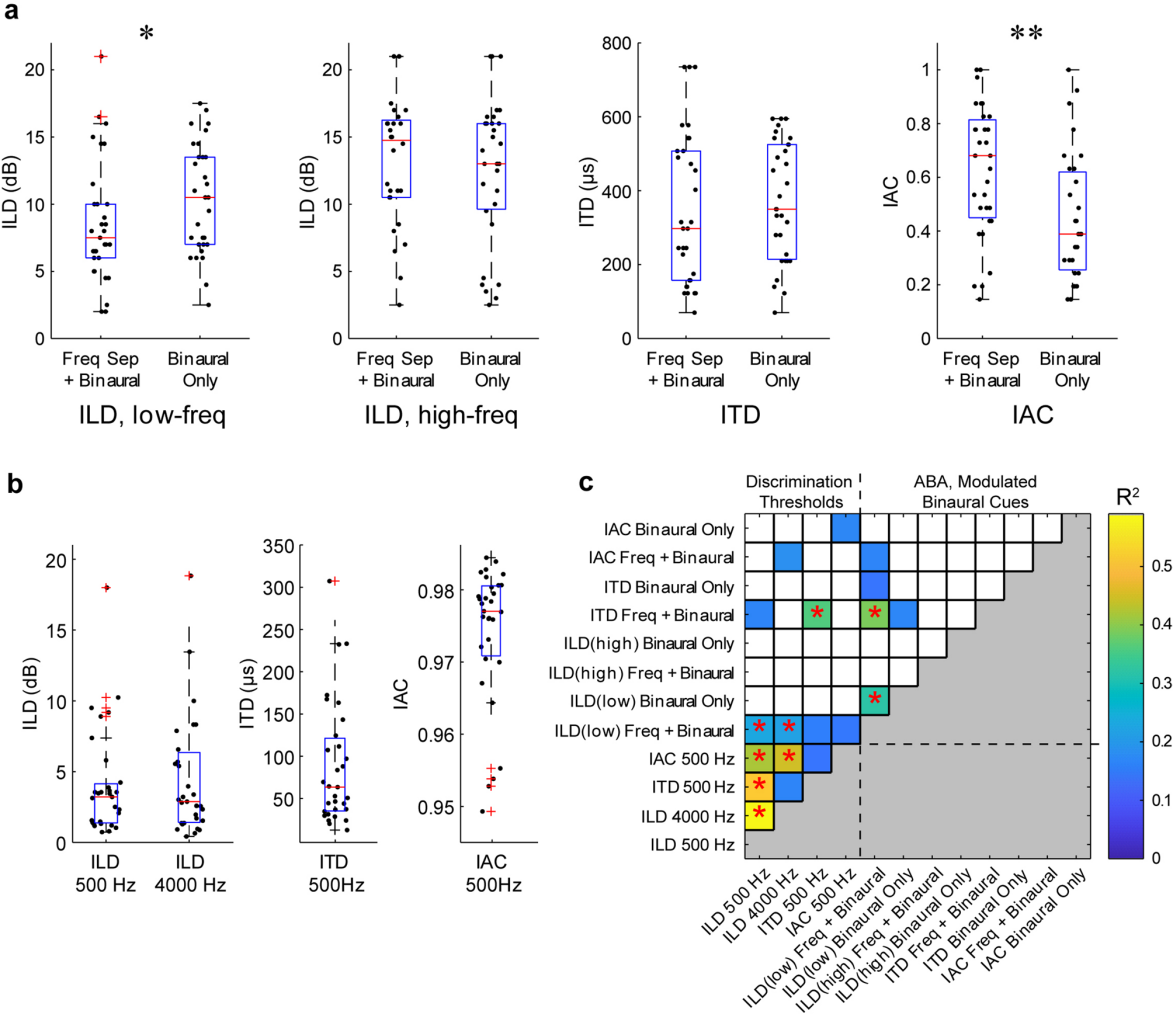
(**a**) Boxplots of binaural cue segregation boundaries for all modulated binaural cue conditions. Asterisks indicate significant differences between conditions (*p* < 0.05). (**b**) Boxplots of discrimination thresholds for ILD low-frequency (panel 1), ILD high-frequency (panel 1), ITD (panel 2), and IAC (panel 3). Outliers indicated with red crosses. **c**) Correlation matrix representing the association between each condition of Experiment 2, including binaural cue discrimination threshold values (left of dashed line) and binaural cue segregation boundaries (right of dashed line). Red asterisks indicate a significant correlation (*p* < 0.05, corrected for multiple comparisons^42^, colored cells indicate correlations with *p* < 0.05 (uncorrected). White cells indicate no association (*p* > 0.05). Note that the color bar is scaled from 0 to the maximum observed correlation value.

Distributions of binaural cue segregation boundaries (Fig. 7a) covered a wider range than the discrimination thresholds (Fig. 7b) for each respective cue. Binaural cue segregation boundaries were significantly wider (larger) for the low-frequency ILD-modulated condition (Fig. 7a, panel 1) without the frequency modulation than with (t_30_ = 2.41, *p* = 0.022, d = −1.7258), meaning that it took a greater difference between the A- and B-components in ILD space to prompt a perceptual switch from integrated to segregated. Similarly, IAC segregation boundaries (Fig. 7a, panel 4) without frequency separation were significantly lower than with frequency separation (t_30_ = 3.29, *p* = 0.0026, d = 0.18), meaning that on average participants required a larger separation in IAC between the A- and B-components to report a segregated percept. No significant differences were observed for the ILD-high frequency and ITD conditions as a function of frequency separation.

#### Statistics of binaural cue segregation boundaries and discrimination thresholds

Observed discrimination thresholds were in a range consistent with the literature apart from a few outliers: on average ILD thresholds were less than 5 dB, ITD thresholds less than 150 µs, and IAC values were greater than 0.96, equivalent to a Δ-IAC (or Δρ) of 0.04^12 ^(Fig. 7b). Figure 7c presents a correlation matrix quantifying the relationship between all conditions, including discrimination thresholds and binaural cue segregation boundaries. Colored cells indicate correlations with a p-value < 0.05 (uncorrected); cells with asterisks indicate significant correlations (*p*< 0.05; corrected for multiple comparisons^42^. Note that when the correlation matrix was isolated to just the four discrimination threshold datasets, all correlations were significant following statistical correction for multiple comparisons, a result consistent with and expected from the literature^12^. Segregation boundaries for the low-frequency ILD with separation showed significant correlation with discrimination thresholds for all conditions. ITD segregation boundaries were significantly correlated with ITD discrimination thresholds and low-frequency ILD segregation boundaries.

#### Perceptual hysteresis

**Fig. 8:**
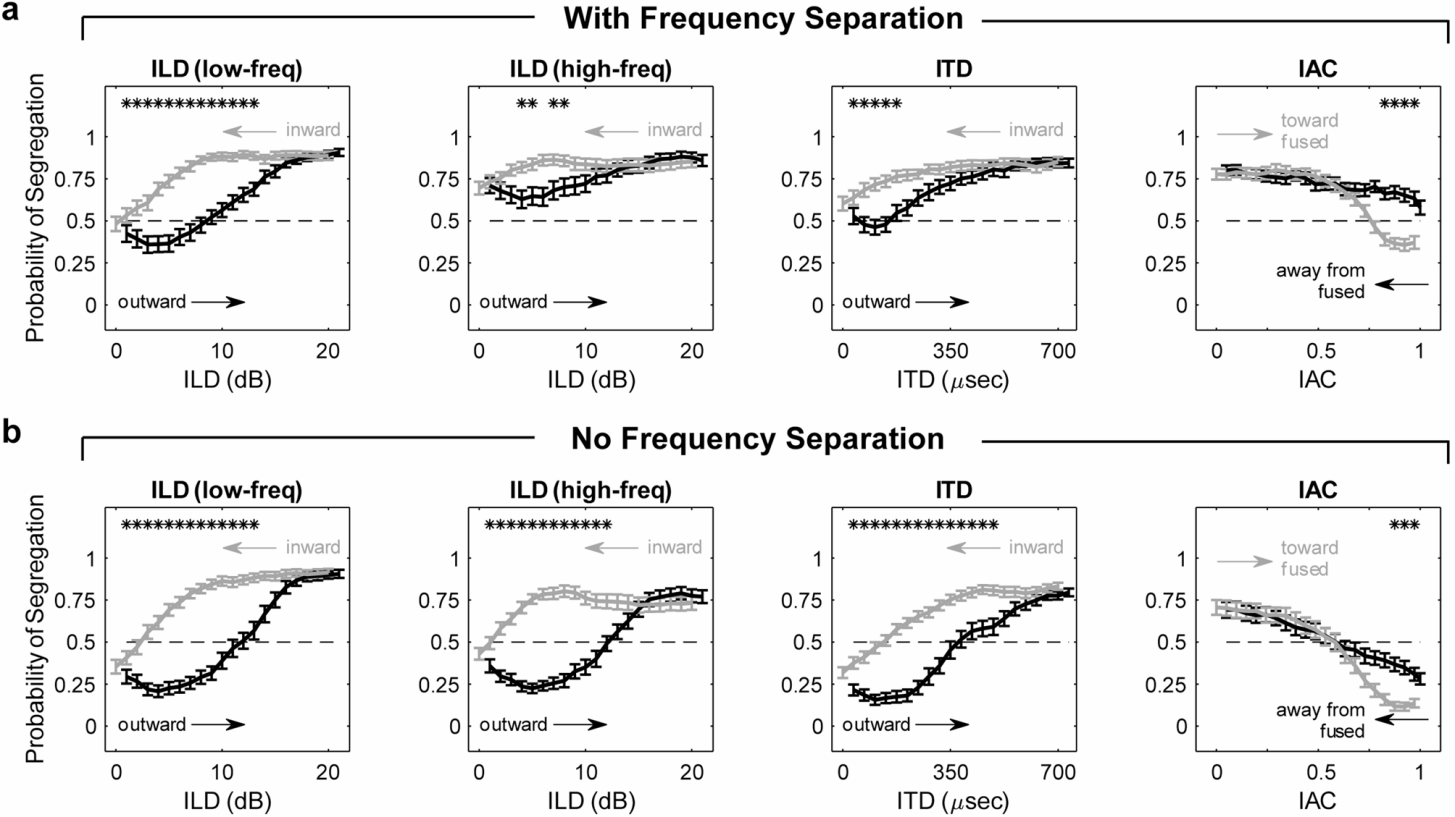
Perceptual Hysteresis. All binaural cue modulated conditions (**a**) with frequency separation and (**b**) without frequency separation. Each panel plots the probability of a segregated percept as a function of the absolute value of the binaural cue, separated by the direction of binaural cue modulation. Black lines correspond to participant responses when the cue was moving outward, or laterally away from the diotic B-component (i.e., the midline). Gray lines represent responses when the cue was moving inward, or towards the diotic B-component (i.e., the midline). Asterisks indicate significant point-to-point differences (paired t-test; *p* < 0.01, corrected for multiple comparisons^42^. Errorbars correspond to the standard error of the mean.

Perceptual hysteresis represents “the enduring influence of the recent past on current perception”^43^. From a cognitive-systems perspective, the ability to sustain a continuous, stable representation of a percept over time is a feature of auditory stream segregation. This phenomenon was exhibited when participant response patterns were analyzed based on the direction of the binaural cue modulation of the A-component relative to the B-component, i.e., moving away from the B, or moving towards the B. For ILD and ITD modulation, the percept may be characterized as moving outward (away from the diotic-midline) or inwards (towards the midline). The IAC can be characterized as moving towards fusion of the A- and B-components, or moving away from fusion, i.e., the B remains at IAC = 1 while the A-components became progressively diffuse (decorrelated). All conditions showed clear evidence of perceptual hysteresis. For example, while moving *outward* the low-frequency ILD with frequency separation condition, tended to be integrated, crossing the equal probability point (0.5) at approximately 8.5 dB ILD (Fig. 8a, panel 1, black line) and at 11.8 dB ILD without the frequency separation (Fig. 8b, panel 1, black line). Conversely, when moving *inward*, a segregated percept tended to be maintained, with the equal probability point crossed at less than 1 and 2.4 dB ILD for those same conditions (Fig. 8a,b, panel 1, grey lines). The same pattern was observed for the high-frequency ILD (Fig. 8, column 2), and the ITD conditions (Fig. 8, column 3). For the IAC modulation, the group average pattern was slightly different. As the A-component moved *away* from the fused diotic-midline point, response probabilities slowly became more segregated (Fig. 8, column 4, black lines). As the A-component converged from diffuse (IAC = 0) to fused (IAC = 1) perception was relatively flat and then steeply decreased towards an integrated percept with a transition point at approximately IAC equal to 0.65 (Fig. 8, column 4, grey lines).

### Discussion

Results of Experiment 2 show a clear influence of modulated binaural cues on auditory stream segregation compared to the diotic, control condition. For all cues tested, binaural cue segregation boundaries were defined by fitting each participants’ responses to a model shape, either an inverse Gaussian for ILD and ITD conditions or an inverse log function for the IAC conditions and estimating the point of transition in lateralized space where perception switched between integration and segregation (Fig. 6). Segregation boundaries were then compared to individuals’ discrimination thresholds, with results showing a mixture of significant associations between the metrics of sensitivity to each cue (Fig. 7). The perceptual hysteresis in Fig. 8 illustrates both the influence of modulated binaural cues while also revealing the plasticity of perception encompassing a wide range of stimuli, and the strength of preceding contextual cues on steam segregation.

#### Effect of frequency separation

Conditions with a frequency separation between the A and B-components elicited a significantly higher proportion of segregated than integrated percept (Fig. 5). This observation can be seen in Fig. 4 at binaural cue levels where the A-component was close to the B (midline-locations). For the frequency-separated conditions, at these binaural cue levels perceptual responses were consistently bistable, akin to the diotic condition, while the no-frequency-separated conditions exhibited substantially more integrated percepts (blue versus orange lines at midline).

The high frequency ILD condition was included in this experiment because that is the frequency range that ILD cues are observed at in the free field. The same parameters were used as with the lower frequencies, 6 semitone center-frequency separation (4000, 5650 Hz) and 1/3rd octave bandwidth, and similar bistability was expected. Compared to the low frequency ILD conditions, one hypothesis is that more segregation would be observed for the high frequency ILD, since those are the most ecologically relevant frequencies for this cue^13^. In contrast a second hypothesis is that there would be less segregation since difference limens increase with frequency^44^. In other words, it should take a greater frequency separation to distinguish between two tokens. Figure 5, however, shows an interaction between the low frequency ILD conditions and the high frequency ILD conditions. *With* frequency separation, significantly more segregation is observed in the high than low frequency ILD conditions. Conversely, *without* frequency separation, significantly less segregation was observed in the high versus low frequency conditions. This indicates that the contrast of the high frequency A- and B-components was more responsible for the increased segregation than the salience of the ILD cues carried by the high frequency A- and B-components. When the frequency separation was removed the amount of segregation was decreased more than in the low-frequency ILD conditions. These results further support the idea that binaural cues are most effective in combination with a frequency-separation-framework and significantly less effective when alone, or static.

#### Individual differences: sensitivity to binaural cues

Here, by modulating binaural cues with a high-resolution step size around the midline (B-component), combined with a traditional discrimination threshold paradigm, we can evaluate individual sensitivity to binaural cues in both the stream segregation domain as well as the psychophysical limitation domain. The distribution of discrimination thresholds observed here (Fig. 7b) is generally comparable to those observed by Spencer et al.^12^ excepting a few elevated thresholds in our dataset. Two experimental differences are likely explanations for those outliers: (1) Spencer et al. instructed participants that the target interval would always be to the left of the standard intervals whereas we retained a random sampling of left and right targets from the standard, making the task more difficult, and (2) they had a participant pool of listeners who returned multiple times and were generally experienced listeners, whereas our study ran the battery of discrimination threshold tests on a participant pool of low listening-experience young adults and only repeated an adaptive track if they expressed confusion or had high inter-trial variability. Nevertheless, within the discrimination threshold dataset, significant correlations were observed between the cues (Fig. 7c).

One additional interest of this study was the investigation of the individual differences in sensitivity to binaural cues in individuals without extensive listening experience. Previous studies have noted extreme variability across this type of listener pool^41,45,46^ leading to speculation that task familiarity may be responsible. By introducing the experimental task as a stream segregation paradigm and familiarizing participants with bistable perception we were able to probe suprathreshold binaural cue sensitivity orthogonally, and in a way that spans the full lateralized range of cues. The full width at half-minimum metric of the ILD and ITD functions (Fig. 6b, panels 1 to 3, dashed lines) was used to quantify sensitivity to the cue because it captures the depth and range of their perceptual profile but is insensitive to bias towards one percept or the other. Comparisons of binaural cue segregation boundaries across cues showed significant correlations. Notably the low-frequency ILD with frequency separation was correlated with both ILD discrimination thresholds, and weaker (uncorrected) correlations with ITD and IAC discrimination thresholds (Fig. 7c). ITD segregation boundaries were significantly correlated with ITD discrimination thresholds and IAC segregation boundaries showed a weak relationship with high frequency ILD discrimination thresholds. Overall, while these two measures are not perfectly aligned, there is substantial evidence supporting the hypothesis that both measures, segregation boundaries and discrimination thresholds, reflect a common underlying mechanism, in contrast to Middlebrooks and Onsan^23^. In other words, listeners who are sensitive to small differences in a binaural cue, are more likely to use that cue to segregate auditory sources.

#### Perceptual hysteresis

Perceptual hysteresis is a known phenomenon characterized by a tendency to retain a percept despite changing sensory input and underscores the challenge of perceptual research due to the instability of perceptual thresholds^43,47^. The alternative to hysteresis is perceptual “resetting” due to an abrupt change in a signals’ properties^1,8^. These are also referred to as positive hysteresis (maintaining percept due to recent history) versus negative hysteresis (switching percept due to recent history)^47^. The observation of hysteresis here (Fig. 8) is evidence that the modulation in binaural cues was sufficiently slow to avoid abrupt resetting of segregation^5,26^, mirroring the results observed by Schadwinkel and Gutschalk (2011)^10^, but fast enough to elicit a percept of relative motion^48^. The counterargument that these results do not reflect true perceptual memory but rather show an effect of subjective inertia or experimental complacency is challenged by the directionality differences in the response patterns. Stated differently, if a uniform response delay was responsible for these results, it would be reflected equally across the stimulus range. Instead, the effect is strongest in the region for each cue that the contrast between the A and B is most ambiguous, i.e., around IAC = 1, indicating that the perceptual memory is weighted higher in those cases than when the cues are very different from each other (e.g., IAC = 0). Combined with the contrast observed between static binaural cue conditions (Fig. 2) and the modulated binaural cue conditions (Fig. 4) these results present clear evidence that binaural cues in the absence of dynamic change have a weak influence on auditory stream segregation but are strongly influential when forced to continuously update.

## General discussion

Spatial separation of speech signals due to spatial release from masking is one of the most studied aspects of auditory scene analysis that focuses on spatial or binaural cues. General spatial awareness in comparison, is much less studied even though it plays a pivotal role in how we monitor and navigate our ever-changing acoustic environment. Here, we asked the question: to what degree does the auditory system rely on binaural cues to build a percept of segregated or integrated auditory streams? The experiments presented here demonstrate that given an otherwise ambiguous, or bistable stimulus, (1) static binaural cues do a poor job of influencing perception, (2) modulated binaural cues strongly influence perception, (3) the degree to which an individual is influenced by modulated binaural cues is related to their discrimination thresholds, and (4) listener responses show strong evidence for perceptual hysteresis (i.e., maintenance) in the presence of changing binaural cues.

### Binaural cues: static versus modulated

In contrast with Experiment 1, the results of Experiment 2 showed a strong influence of binaural cues on stream segregation, the only difference being whether the cues were static or modulated. In Experiment 2, the conditions with frequency separation effectively demonstrated perceptual bistability whenever the A and B-components were similar (Fig. 4, blue lines), same as in the diotic (control) condition. The active modulation of the A-component away from the diotic B, counteracted the bistable nature of the intermediate, 6-semitone, frequency separation and influenced individuals’ percepts towards more segregated. One way to interpret the ineffectiveness of static binaural cues to influence stream segregation has to do with tonotopic (cochleotopy) organization of the auditory system. From the point of transduction of acoustic energy to neural impulses, tonotopic organization (i.e., cochleotopy) provides the framework that other sound features are incorporated within, and has a very strong influence on auditory stream segregation^9,49^. Conceptually this process can be described by partially overlapping neural representations of each frequency (A- and B-components), where over the course of time neural dynamics related to mutual inhibition and adaptation drive perception to spontaneously alternate when presented with ambiguous, or bistable stimuli^10,50–52^. In the context of Experiment 1, whether binaural cues similarly evoke partially overlapping populations appears to be inconsequential, as the frequency separation took precedent, and the binaural cues had minimal impact.

In the modulated binaural cue conditions, the relative lateralized difference between the A- and B-components forces the percept to continuously update and becomes the dominant cue over time, comparable to Byrne et al.^9^ who observed perceptual entrainment due to frequency modulation. Schadwinkel and Gutschalk^10^ leveraged this forced perceptual update in a paradigm similar to that presented here, by setting the A-component of an ABBB quadruplet at −687 µs and slowly modulating the B-components by 41.76 µs every 4 or 6 quadruplets (in separate trial-types) towards the midline, switching lateral direction after 32 s. Behavioral results showed that the onset of segregation mostly occurred on the rising ITD difference (B moving away from the A-component) while a return to integration occurred on the decreasing ITD difference, with variability over a wide range > 10 s, showing comparable segregation boundaries and perceptual hysteresis as the results reported here (Figs. 5c and 7a, 3rd panel). As the primary focus of that experiment^10^ was measurement of the cortical BOLD (blood oxygenation level-dependent) signal during perceptual reversals, this paradigm had the benefit of loosely constraining the perceptual dynamics of perception with the benefit of not tightly locking perception and switches-in-perception to a specific lateralized position. In summary, the difference in effectiveness of static versus modulated binaural cues to influence perception is due to the interaction between frequency and time. When spatial cues are uninformative over time (unchanging), frequency is the dominant feature driving stream segregation. When spatial cues are informative across time, their impact on perception is much stronger.

## Conclusion

The results of this study characterize the efficacy of binaural cues (ILD, ITD, and IAC) to influence auditory perception using a framework that allowed for the separation of stimulus effects related to static versus modulated cues and frequency separation. Modulated binaural cues were significantly more influential for evoking a segregated percept at lateral positions than static cues at comparable ILD and ITD positions. Comparison of behavioral response patterns for stimuli with and without a 6-semitone frequency separation showcase the primary contextual role of frequency for the interpretation of binaural cues by the auditory system, while emphasizing the interaction of the two when the binaural cues are modulated. On an individual basis, the influence of binaural cues on stream segregation is at least partially related to the discrimination threshold of that cue for that individual.

## Data Availability

Data are available at the Center for Open Science: [https://osf.io/jqgxf/overview? view\_only=63fd761b205147f6aee9908d736dfeda](https:/osf.io/jqgxf/overview? view_only=63fd761b205147f6aee9908d736dfeda).
